# The Teaching and Learning Cultural Competence in a Multicultural Environment (CCMEn) Model

**DOI:** 10.3390/nursrep10020019

**Published:** 2020-12-14

**Authors:** Isabel Antón-Solanas, Margarida Coelho, Isabel Huércanos-Esparza, Valérie Vanceulebroeck, Indrani Kalkan, Raul Cordeiro, Nuran Kömürkü, Teresa Soares-Coelho, Nadia Hamam-Alcober, Shana Dehaes, Antonio Casa-Nova, Lucía Sagarra-Romero

**Affiliations:** 1 Research Group GENIAPA, Department of Physiatry and Nursing, University of Zaragoza, C/Domingo Miral s/n, 50009 Zaragoza, Spain; 2 Instituto Politécnico de Portalegre, School of Education and Social Science, Praça do Município, 77301-110 Portalegre, Portugal; margco@ipportalegre.pt (M.C.); teresa.coelho@ipportalegre.pt (T.S.-C.); 3 Faculty of Health Sciences, Universidad San Jorge, Autovía A-23. Km 199, 50830 Villanueva de Gállego, Spain; ihuercanos@usj.es (I.H.-E.); lsagarra@usj.es (L.S.-R.); 4 Department of Nursing, AP University College, Lange Nieuwstraat 101, 2000 Antwerpen, Belgium; valerie.vanceulebroeck@ap.be (V.V.); shana.dehaes@ap.be (S.D.); 5 Faculty of Health Sciences, Istanbul Aydin University, Sefaköy-Küçükçekmece 38, 34295 Istanbul, Turkey; indranikalkan@aydin.edu.tr (I.K.); nurankomurcu@aydin.edu.tr (N.K.); 6 Instituto Politécnico de Portalegre, School of Health Sciences, Praça do Município, 77301-110 Portalegre, Portugal; raulcordeiro@ipportalegre.pt (R.C.); casanova@ipportalegre.pt (A.C.-N.); 7 Servicio Aragonés de Salud, Women’s and Children’s Hospital, Paseo Isabel la Católica, 1-3, 50009 Zaragoza, Spain; nhamam@salud.aragon.es

**Keywords:** cultural competency, nursing education, educational model, minority health

## Abstract

Background: Within the European higher education context, students and lecturers are encouraged to engage in teaching and learning activities abroad. This frequently involves using a second language and being exposed to students and lecturers from culturally different backgrounds. Objective: To design a model for teaching and learning cultural competence in a multicultural environment (CCMEn). Design: Theory development from empirical experience, research, and scholarly works. Method: This model was developed based on our experience of teaching and learning cultural competence in a multicultural environment in a nursing education context; it rests on three pillars, namely, Coyle’s Content and Language Integrated Learning educational approach, the concept of social and emotional learning, as defined by the Collaborative for Academic, Social and Emotional Learning, and the existing literature surrounding teaching and learning cultural competence in higher education. Results: The CCMEn model is intended to guide the process of teaching and learning cultural competence in a multicultural environment through the use of a second language and has been adapted from existing educational approaches and theory. Conclusion: Teaching and learning in multilingual and multicultural contexts in Europe is becoming more common. Students who learn alongside students and teachers from different cultural backgrounds need to be supported from an academic, linguistic and socioemotional perspective. We believe that the CCMEn model can serve as a guide to enhancing student learning in this context.

## 1. Introduction

According to the European Commission [1], “social inclusion is at the core of the European Social Model and European values enshrined in the Lisbon Treaty”. Nevertheless, in the past few years, social exclusion and inequality have become a major concern in European society. A changing society, with free movement of citizens within the European Union (EU), distant parts of our globe becoming more accessible, and an increasingly multicultural population, are factors impacting on healthcare, highlighting the need for cultural competence among health professionals [2,3]. European higher education institutions (HEIs) have a responsibility to address these issues in the curricula through the reinforcement of fundamental rights and democratic values, the promotion of social inclusion, nondiscriminatory active citizenship and critical thinking, and the integration of social, civic and transcultural competence. 

There is a wealth of models and approaches for implementing cultural content in the nursing curricula, mainly emerging from Leininger’s theory of cultural care diversity and universality [4]. However, we have observed that they each offer partial solutions when implemented in a multicultural, multilingual learning environment as part of a short intensive course. Specifically, some models, such as Campinha-Bacote’s [5] Process of Cultural Competence and Jeffrey’s [6] Cultural Competence and Confidence Model, include a practical component which requires students to demonstrate a degree of cultural skill. This is achieved by exposing the students to cultural encounters with patients from diverse cultural backgrounds. Whereas it is desirable to give students the opportunity to put newly acquired knowledge into practice [7], it is not always feasible to integrate a practical component into a short intensive course, due mainly to a lack of time and resources. Another frequent problem with pre-existing models, such as Papadopoulos Tilki and Taylor’s [8] model of Cultural Competence, is the inclusion of a final stage or component entitled cultural competence. Whereas the acquisition of cultural competence is, ultimately, the desired outcome, we do not expect our students to achieve any degree of cultural competence during the course, but rather to develop knowledge, skills and attitudes which may, in future, allow them to gradually become more able to develop a culturally mindful and safe practice. Finally, no pre-existing educational models for the teaching and learning of cultural competence in a multicultural environment, in a nursing educational context, have been identified in the literature. There is an interesting precedent in Banks’ [9] five dimensions of multicultural education, which seek to facilitate the conceptualisation and implementation of multicultural education by “transforming the culture and values of the organisation to ensure fair and equitable treatment for all” [10]. We fully agree with this idea and argue that cultural difference must be celebrated and acknowledged in order to foster the attainment of cultural knowledge, skills and attitudes among culturally diverse students. However, Banks’ model was designed to be implemented on a much larger, organisational level. 

In addition to the above, our student and teacher populations are very diverse not only in terms of nationality and language but also in terms of religion, age, culture and educational context. If not addressed, this diversity impacts not only the teaching and learning process but also the process of communication and social interaction between teachers and students and between the students. In particular, frequent problems with our student population included difficulties with the English language, social integration and connectedness, and even some degree of stress and anxiety [11,12]. 

**Figure 1:**
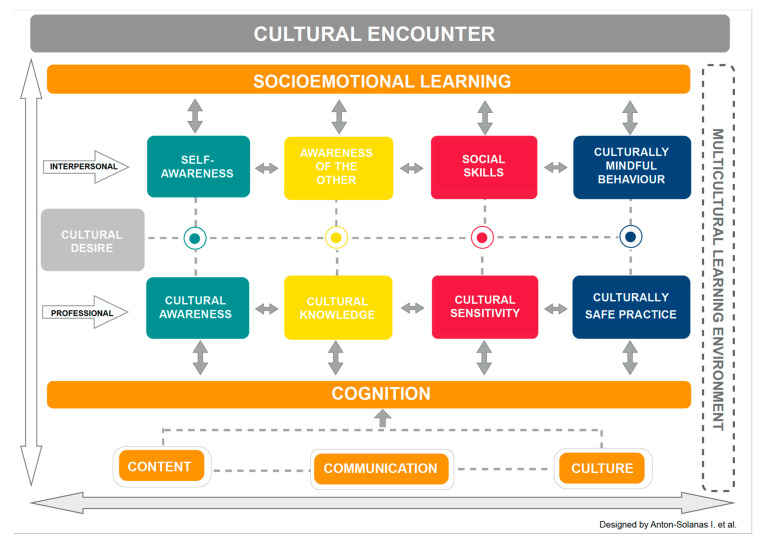
The Cultural Competence in a Multicultural Environment (CCMEn) Model.

This paper is part of a larger EU-funded project entitled Transcultural Nursing: A European Priority, a Professional Responsibility (TC-Nurse) [13] which focuses on cultural, linguistic and religious diversity and promotes ownership of shared values, equality, nondiscrimination and social inclusion through education and training at the higher education level. As part of this project, 24 undergraduate nursing students and eight lecturers from four European universities, namely, Universidad San Jorge (coordinator, Spain), Instituto Politécnico de Portalegre (Portugal), AP Hogeschool (Belgium) and Istanbul Aydin University (Turkey), come together for a week once a year to learn about transculturality and cultural competence. All of our students and staff speak English as a second language and come from four European countries. They are culturally diverse in terms of their religion, ethnicity, social class, clinical work experience and exposure to people from a minority cultural background. Culturally diverse students must receive adequate support when learning in an international environment [14,15]. Therefore, we have designed a model for the teaching and learning of cultural competence in a multicultural environment (CCMEn), with the aim of addressing these issues and improving the teaching and learning experience of culturally diverse students and teachers based on our experience and an extensive review of the literature (Figure 1).

## 2. The CCMEn Model

The CCMEn model is intended to guide the process of teaching and learning cultural competence in a multicultural environment through the use of a second language and has been adapted from existing nursing education approaches and models. The CCMEn model rests on three pillars. First, we have adopted a Content and Language Integrated Learning (CLIL) approach, in order to support student learning of content through a second language [16]. Second, in order to address the problem of communication and social interaction, we have integrated a layer of social and emotional learning competencies [17], which are explicitly integrated into the teaching plans (Table S1) and can be tailored to the characteristics of our student population. Third, we have designed a five-stage process for the teaching and learning of cultural competence in a multicultural environment based on the existing literature [5,6,8,9].

### 2.1. A CLIL Approach

It is rare for culturally diverse students to share a common mother tongue, especially in the context of European higher education. Thus, learning in a multicultural environment, with culturally diverse students and teachers, must often be achieved through the use of a second language, a lingua franca. In order to address the challenges of the novel multicultural and multilingual educational settings, and the specific needs of the different stakeholders involved in the process, a CLIL approach has been adopted to support both content and language learning through a second language [18]. Coyle’s [16,19] 4Cs Framework is based on the tenet that the strengthening and development of a learner’s conceptual understanding involve social, cultural, linguistic and cognitive processes and offers a comprehensive theoretical and methodological foundation for planning CLIL lessons, designing activities and constructing materials. The core elements or constructs of the 4 Cs Framework are as follows:

- Content: What the students need to know.
- Communication: The language skills that the students need to have in order to work on the content both autonomously and in the classroom.
- Culture: The students’ cultural heritage that shapes their experiences, personal values, reflective processes and behaviours.
- Cognition: The thinking processes that the students need to use in order to engage with and understand course content.

**Figure 2:**
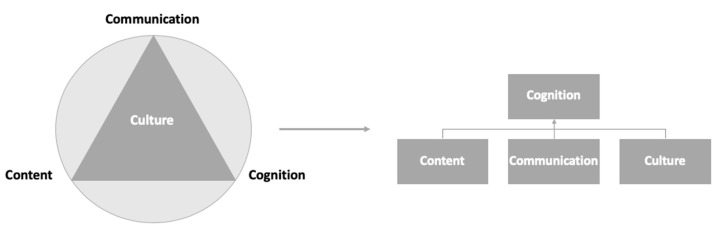
Rearrangement of the 4 Cs: cognition, content, communication and culture.

The CCMEn model incorporates all four constructs, but they have been rearranged with the aim of giving the Culture element a much more prevalent role (Figure 2). In the original 4Cs Framework, Content, Cognition and Communication form the basis of content and language integrated learning, whilst Culture permeates the other elements, allowing the students to develop intercultural understanding and reinforcing learning [19]. According to Meyer [20], “realizing that other cultures tend to see things differently, have different values and beliefs, is one of the most valuable experiences that CLIL may offer”. However, in practice, the cultural dimension has not been properly exploited yet [20]. In the CCMEn model, Content, Communication and Culture are explicitly integrated into the teaching plan and underpin the design of the intended learning outcomes (ILOs), teaching and learning activities and assessment tasks, all of which are integrated into the fourth C, Cognition (Table S2). The rationale for this change is twofold: the students’ culture must be carefully considered when teaching and learning in a multicultural environment and it must be integrated into the teaching plans to promote meaningful cultural encounters. 

### 2.2. Socioemotional Learning

Adding a further theoretical perspective to frame Coyle’s 4 Cs model, the CCMEn model integrates a fifth element into the multicultural learning environment: socioemotional learning. Socioemotional learning is defined as the process by which individuals acquire the knowledge, learn to understand and manage their emotions, maintain positive relationships, make responsible decisions and demonstrate a caring attitude and concern for others [21,22]. Traditionally, in academic settings, socioemotional skills are frequently and commonly displaced to minor roles in favour of the development of the cognitive domain and academic achievement [23]. However, social and emotional competence is also crucial for adult learners, particularly when teaching and learning take place in a challenging or “emotional environment” [24]. This is the case when students and teachers engage in a process of teaching and learning in a multicultural environment through the use of a second language. In our experience, undergraduate nursing students do not necessarily have the knowledge and skills necessary to perform well in a multicultural learning environment. This phenomenon has also been observed in preceding multicultural teaching and learning experiences. [25]. In particular, some of our students expressed difficulties communicating with students who were perceived as being different and felt uneasy when undertaking group work and speaking in public. Thus, we propose that socioemotional ILOs, teaching and learning activities and assessment tasks are designed alongside cognitive ILOs, teaching and learning activities and assessment tasks, and are integrated into the teaching plans. We argue that careful consideration of the socioemotional skills and attitudes needed to effectively engage in the cognitive teaching and learning activities will contribute to creating a positive learning environment in which students from different cultural contexts can feel safe and comfortable. Thus, student learning will occur in parallel at two levels, namely, cognitive and socioemotional, and will be supported by three pillars emerging from the CLIL approach, namely, content, communication and culture. Teachers must be mindful of this when designing the teaching plans and must facilitate student learning at two levels, namely, interpersonal—through the acquisition of knowledge, skills and attitudes, leading to the display of a culturally mindful behaviour, both towards their fellow students and the facilitators—and professional—through the acquisition of knowledge, skills and attitudes leading to a culturally safe professional nursing practice. 

### 2.3. A Five-Stage Process

Surrounded and supported by these five constructs: Content, Communication, Culture, Cognition and Socioemotional learning, and promoted through constant cultural encounters, the acquisition of knowledge, skills and attitudes leading to a culturally mindful and safe nursing practice, according to the CCMEn model, occurs in five phases or stages: (1) cultural desire, (2) self-awareness/cultural awareness, (3) awareness of the other/cultural knowledge, (4) social skills/cultural sensitivity and (5) culturally mindful behaviour/culturally safe practice. 

The first stage of the CCMEn model is cultural desire. Cultural desire is defined by Campinha-Bacote [25] as the motivation or desire of nurses to actively engage in the process of becoming culturally competent. It requires a personal motivation to develop new skills and acquire new knowledge, leading to culturally mindful and safe personal behaviour and a professional nursing practice [26]. From an educational perspective, this first stage must include activities that allow the culturally diverse students and facilitators to begin to develop a trusting relationship with each other; for example, ice-breakers and group activities, which may include gamification. These activities should focus primarily on areas of common interest and focus on similarity rather than cultural differences. The content should focus on contemporary issues relating to transculturality and nursing care, preferably applicable to the students’ sociocultural context. For instance, for European students, the Syrian crisis and ever-growing number of migrants and refugees seeking asylum in the EU are highly relevant and current themes. The establishment of interpersonal relationships in the classroom may be limited by the fact that human interaction is somewhat constrained by the teaching plan. Thus, it may be useful to promote a freer form of cultural encounter between the students through sociocultural activities in which facilitators may or may not participate. 

The second stage of the CCMEn model is self-awareness/cultural awareness. From a socioemotional perspective, the students should be encouraged to reflect on their personal identity both as individuals and as professionals. It may be useful to discuss the students and teachers’ educational culture, which is understood as the framework in which educational activities take place [27], and promote self-reflection and interpersonal sharing through teaching and learning activities [5]. In addition, the students should begin to identify their own emotions when interacting with students from other cultural backgrounds by recognising how being in a multicultural environment may influence their behaviour and demonstrate self-confidence. From a cognitive point of view, students should be encouraged to reflect on the values and beliefs shaping their identity as nurses and caregivers. Concepts such as ethnocentricity, stereotyping, cultural identity and cultural humility should be addressed at this point [5,8,28]. 

The third stage is awareness of the other/cultural knowledge. At this stage, the students should now begin to successfully manage their own emotions, thoughts and behaviours. They should feel comfortable in the multicultural learning environment and demonstrate discipline and motivation to learn alongside and collaborate with fellow diverse students. From a cognitive perspective, the students will be given opportunities to discuss and compare health-related beliefs, practices and behaviours, and biological, psychological, sociopolitical and anthropological characteristics of selected cultural groups. They will develop an understanding of current issues affecting patient health outcomes, including limited access to healthcare, health inequalities and patient empowerment [26]. Case scenarios may be used to illustrate these concepts and allow the students to apply new knowledge [29]. 

The fourth stage focuses on social skills/cultural sensitivity. From a socioemotional point of view, classroom activities should encourage the students to take the perspective of and empathise with others, showing respect and appreciation of the other person’s viewpoints. Moreover, special attention should be paid to the communication process between the students themselves and between the students and the facilitators. At this stage, the students should be able to communicate clearly with each other using a variety of both verbal and nonverbal strategies and resources. These may include active listening, giving and receiving feedback, conflict negotiation abilities, relationship building and teamwork. These values and attitudes are easily transferrable and applicable to the clinical context. For example, lessons learnt while working and learning alongside students from diverse cultural backgrounds may be useful when working in a multicultural team of healthcare professionals. From a cognitive perspective, students should discuss the nature of the nurse–patient relationship in their own cultural context and reflect on the use of power in healthcare. Concepts such as trust, respect, acceptance and negotiation and their impact on the health and wellbeing of culturally diverse patients, should be introduced. It is also important to discuss communication barriers and promoters, including language difficulties, misunderstandings and intercultural communication [30,31]. 

The fifth and final stage is culturally mindful behaviour/culturally safe practice. At this stage, the students will be expected to demonstrate both culturally mindful behaviour towards each other and culturally safe nursing practice. We cannot expect students to become culturally competent individuals and nursing professionals at this point [4,32]. However, we can assess the learning of socioemotional and cognitive cultural knowledge, skills and attitudes through both formative and summative assessment. Facilitators should measure and assess student learning through both quantitative and qualitative means. Problem-solving activities, role play, clinical simulation and group work may be useful to evaluate the students’ ability to identify and solve problems, analyse situations, resolve conflict and, in sum, demonstrate safe nursing practice. Other useful resources include validated questionnaires such as Chen’s Intercultural Sensitivity Scale [33] and Papadopoulos’ Cultural Competence Assessment Tool [34]. Specific qualitative techniques, including personal and group interviews, observation and debriefing sessions, may generate information about the students’ cognitive and socioemotional learning experience, both individually and as a group. The self-assessment of the SEL competencies questionnaire may be used to measure the students’ perceived level of socioemotional competence [35]. It is important to highlight that the summative assessment of student learning must be supported through formative assessment and feedback throughout the course. Further, student assessment must be aligned to teaching and learning activities and intended learning outcomes, as proposed by [36]. 

## 3. Discussion

The CCMEn model has been designed to guide the process of teaching and learning cultural competence in a multicultural environment through the use of a second language in a nursing education context. It is intended to provide opportunities for interpersonal and professional learning of the knowledge and skills necessary to develop a culturally safe nursing practice and a culturally mindful personal behaviour. The CCMEn model is but a small part in a life-long journey towards cultural competence [32,37]. The students should not be expected to suddenly become culturally competent nurses. Instead, they should be allowed to make and recognise their mistakes, gradually enlarging their knowledge on different cultures and patient groups and refining their behaviour towards not only culturally diverse patients but also colleagues and other people from outside the health system [4]. Furthermore, the process of learning cultural, social and emotional competencies is not linear but will require the learner to shift back and forth from one phase to another, depending on factors such as personal and professional experiences and training, and can be reinitiated as many times as necessary.

As content teachers, facilitators should not necessarily be expected to be able to implement a CLIL approach when teaching through a second language. Instead, we suggest that specific training is provided to ensure that they are able to integrate language and content learning when planning and delivering their sessions [38,39].

It is important to emphasise that the CCMEn model was designed for use in a multicultural atmosphere in a nursing education context, through the use of a second language, and can potentially contribute to improving teaching and learning in multicultural and multilingual contexts. However, it could easily be adapted for use in less diverse contexts and through the students’ mother tongue. It should not be forgotten that cultural diversity is not just about race and ethnicity but can also be found in age, gender, sexual orientation, religion, and even professional background [30,40]. 

Finally, we recommend that a programme of sociocultural activities, leading to the continuation of cultural encounter, be designed alongside the teaching plan [5]. Facilitators should clearly state the ground rules before the start and ensure that all the students feel safe and comfortable within the multicultural learning environment. For example, it is important to be mindful of the fact that most of the students will not be native English speakers and some may find it challenging to adopt an active role in the classroom; therefore, working in small, mixed groups may be a good idea. 

## 4. Conclusions

Teaching and learning cultural competence in a multicultural environment presents incredible opportunities for learning and some difficulties, which we have tried to address in the CCMEn model. Specifically, the fact that both teachers and students come from different cultural backgrounds creates opportunities for learning that emerge from both classroom activities and constant cultural encounters occurring between the students, the students and the teachers, and both inside and outside the classroom [41,42]. However, content learning has to be supported through language learning when the students are not able to speak the language of instruction as a first language [43]. In our model, this was achieved by introducing and implementing a CLIL educational approach. Additionally, we added a layer of social and emotional learning in order to address the difficulties posed by the multicultural learning environment. In order to simplify the design of the teaching plans, we designed a five-phase model in which socioemotional and cognitive teaching and learning can occur simultaneously. We believe that the CCMEn model can be adapted to other educational backgrounds and can potentially contribute to improving teaching and learning in multicultural and multilingual contexts. It must be taken into account that teaching and learning in a multicultural (and, frequently, also multilingual) environment has become a reality in the European higher education context as a result of the implementation of policies such as the Bologna declaration and the internationalisation of European curricula [44], which encourages both teachers and students to engage in academic exchanges and placements abroad. 

## Supplementary Materials

The following are available online at https://www.mdpi.com/2039-4403/10/2/19/s1. Table S1: Teaching plan; Table S2: Example of a teaching plan using the CCMEn model.
