# Supplementary material for: The Teaching and Learning Cultural Competence in a Multicultural Environment (CCMEn) Model
Source: Nurs Rep. 2020 Dec 14;10(2):154–63. doi: 10.3390/nursrep10020019 (PMC8608070; doi:10.3390/nursrep10020019)
Supplement: Supplementary file 1 [file nursrep-10-00019-s001.zip › Example of a teaching plan.docx]

**Table 2.** Example of a teaching plan using the CCMEn model.

| **CCMEn model stage** | Stage 4: Social skills/Cultural sensitivity |
| --- | --- |
| **Session title** | Defining cultural sensitivity (includes an ice-breaker exercise: “I am, but I am not”) |
| **Facilitator/s name/s** |  |
| **Time** | 40 minutes |

| **CONTENT** | **COMMUNICATION** | | **CULTURE** |
| --- | --- | --- | --- |
| 1. **Session aims:**   To introduce the concepts of misconception and stereotype.  To define and contextualise the concept of cultural sensitivity.   1. **Resources:**    - PowerPoint presentation.    - 24 A-4 size sheets of paper (one per student) and pens.    - Flip-chart paper and markers (various colours).    - Scaffolding materials.    - It would be useful for the students to have access to one computer per group. | 1. **Language *of* learning:**   Ice-breaker: Vocabulary about personal characteristics and stereotypes applicable to the students.  Cultural sensitivity: Frequently used terms to define cultural sensitivity and related vocabulary.   1. **Language *for* learning:**   Ice-breaker: Language to express feelings and emotions in relation to misconceptions and stereotypes.  Cultural sensitivity: Formal terms to write a definition of cultural sensitivity; the students will have a list of key terms, but they will need to use healthcare related vocabulary as well.   1. **Language *through* learning:**   Students will be encouraged to reflect on, and express meaning related to the module’s content in activities of:   - Expressing opinions and emotions about prejudice and stereotype. - Defining cultural sensitivity. - Matching a definition of cultural sensitivity with an image. | | **Culture-specific perspectives and examples:**  Ice-breaker: The students will have an opportunity to discuss stereotypes and misconceptions pertaining to all the cultures represented in the room in safe atmosphere.  Cultural sensitivity: Definitions of cultural sensitivity proposed by authors from a variety of areas of expertise and nationalities have been identified, and will be discussed with the students, in order to demonstrate that cultural competence in nursing is a transcultural concept, which applies to each and every one of them. |
| **COGNITIVE COMPETENCIES** | | **SOCIAL-EMOTIONAL COMPETENCIES** | |
| 1. **Cognitive Intended learning outcomes (C-ILOs)**   By the end of this session the students will be able to:  C-ILO1: Identify misconceptions and stereotypes about themselves and about other students from the same or a different cultural background.  C-ILO2: Formulate an original definition of cultural sensitivity using key terms relating to the term.  C-ILO3: Describe an image using key terms in relation to cultural sensitivity using cooperative learning groups.  C-ILO4: Read existing definitions of cultural sensitivity.   1. **Teaching and learning activities:**   Ice-breaker: “I am, but I am not”   - Each student should fold a piece of paper in half to create two separate columns as follows. The final phrase will read “I am _____, but I am not _____.” - Each student should fill in the first blank with an identifier, such as their race, religion, etc. and the second with a stereotype about that group which is not true of them (whether the stereotype is positive or negative). For example: “I am Spanish, but I cannot dance flamenco.” - Once everyone has written at least 3 statements, they share them with the rest and have an open and respectful discourse on stereotypes.   Defining cultural sensitivity:   - The students will be given 20 key words used by nurse researchers in the past to define cultural sensitivity. In small groups, they must rate them in relation to their relevant to cultural sensitivity. - Next, they must choose at least 4 of them and create an original definition of cultural sensitivity. - Finally, the students must select one picture/image to represent their original definition of cultural sensitivity and explain to rest of the group what the image represents.  1. **Assessment**   Assessment will be formative and will be delivered by the facilitators throughout the activity. | | 1. **Social-Emotional Intended learning outcomes (SE-ILOs)**   By the end of this session the students will be able to:  SE-ILO1: Identify and communicate their own emotions in relation to common misconceptions and prejudice about their own culture.  SE-ILO2: Cooperate with their peers from the same or different cultures in order to complete a task.  SE-ILO3: Engage with the facilitators and their peers, and communicate their ideas and feelings/emotions appropriately.   1. **Teaching and learning activities** 2. Ice-breaker: “I am, but I am not” 3. Once the students have identified common misconceptions and prejudices about themselves, they will share them with the rest of the class and have an open discussion about how they make them feel. 4. **Assessment**   Assessment will be formative and will be delivered by the facilitators throughout the activity. | |
