# Supplementary material for: The Teaching and Learning Cultural Competence in a Multicultural Environment (CCMEn) Model
Source: Nurs Rep. 2020 Dec 14;10(2):154–63. doi: 10.3390/nursrep10020019 (PMC8608070; doi:10.3390/nursrep10020019)
Supplement: Supplementary file 1 [file nursrep-10-00019-s001.zip › Teaching plan.docx]

**Table S1.** Teaching plan template.

| **CCMEn model stage** |  |
| --- | --- |
| **Session title** |  |
| **Facilitator/s name/s** |  |
| **Time** |  |

| **CONTENT** | **COMMUNICATION** | | **CULTURE** |
| --- | --- | --- | --- |
| Include:   - **Session aims** - **Resources**   Audio, video, marker pens, post-it notes, etc. | Include:   - **Language *of* learning**   Topic specific grammar and vocabulary.   - **Language *for* learning**   Language needed to operate in the learning environment –i.e. explain, justify, discuss.   - **Language *through* learning**   Teaching and learning activities (T&L) conducive to new language – i.e. online searching, presenting evidence, discussing evidence. | | Include:   - **Culture-specific perspectives and examples**   Consider the audience’s cultural background (i.e. previous experiences, personal values, reflective processes and behaviours, learning styles) and its impact on the learning process. |
| **COGNITIVE COMPETENCIES** | | **SOCIOEMOTIONAL COMPETENCIES** | |
| 1. **Cognitive Intended learning outcomes (C-ILOs)**   By the end of this session the students will be able to:  C-ILO1  C-ILO2  C-ILO3  C-ILO4   1. **Teaching and learning activities** 2. **Assessment** | | 1. **Socioemotional Intended learning outcomes (SE-ILOs)**   By the end of this session the students will be able to:  SE-ILO1  SE-ILO2  SE-ILO3  SE-ILO4   1. **Teaching and learning activities** 2. **Assessment** | |
